# Multi-label dental disorder diagnosis based on MobileNetV2 and swin transformer using bagging ensemble classifier

**DOI:** 10.1038/s41598-024-73297-9

**Published:** 2024-10-24

**Authors:** Yasmin M. Alsakar, Naira Elazab, Nermeen Nader, Waleed Mohamed, Mohamed Ezzat, Mohammed Elmogy

**Affiliations:** 1 Information Technology Department, Faculty of Computers and Information, Mansoura University, Mansoura, 35516 Dakahlia Egypt; 2 Computer Science Department, Faculty of Computers and Information, Mansoura University, Mansoura, 35516 Dakahlia Egypt; 3 Directorate of Health in Dakahilia, Ministry of Health and Population, Cairo, Egypt

**Keywords:** Dentistry, MobileNetV2, Swin transformer, Annotation, Deep learning, Feature extraction, Computer science, Information technology, Electrical and electronic engineering

## Abstract

Dental disorders are common worldwide, causing pain or infections and limiting mouth opening, so dental conditions impact productivity, work capability, and quality of life. Manual detection and classification of oral diseases is time-consuming and requires dentists’ evaluation and examination. The dental disease detection and classification system based on machine learning and deep learning will aid in early dental disease diagnosis. Hence, this paper proposes a new diagnosis system for dental diseases using X-ray imaging. The framework includes a robust pre-processing phase that uses image normalization and adaptive histogram equalization to improve image quality and reduce variation. A dual-stream approach is used for feature extraction, utilizing the advantages of Swin Transformer for capturing long-range dependencies and global context and MobileNetV2 for effective local feature extraction. A thorough representation of dental anomalies is produced by fusing the extracted features. To obtain reliable and broadly applicable classification results, a bagging ensemble classifier is utilized in the end. We evaluate our model on a benchmark dental radiography dataset. The experimental results and comparisons show the superiority of the proposed system with 95.7% for precision, 95.4% for sensitivity, 95.7% for specificity, 95.5% for Dice similarity coefficient, and 95.6% for accuracy. The results demonstrate the effectiveness of our hybrid model integrating MoileNetv2 and Swin Transformer architectures, outperforming state-of-the-art techniques in classifying dental diseases using dental panoramic X-ray imaging. This framework presents a promising method for robustly and accurately diagnosing dental diseases automatically, which may help dentists plan treatments and identify dental diseases early on.

## Introduction

Ignoring dental issues can be a serious mistake. Although gum disease and cavities cause terrible damage to the teeth, the harm can go much farther^1,2^. Research indicates that dental problems are associated with significant health problems, such as heart attacks and heart disease. Dental illnesses are not to be taken lightly; instead, they are common and dangerous for individuals of all ages and socioeconomic backgrounds^3,4^. These infections can cause excruciating pain, humiliation, and even death by destroying the teeth and gums. To stop these issues before they have a more significant negative impact on health, early detection through dental imaging is essential^5^.

Dental image analysis is essential for detecting and diagnosing oral and dental diseases^6,7^. Due to its ability to aid in the detection of anomalies in the structures of teeth and help the dentist in interpreting different types of problems related to teeth, including the tooth numbers and related diseases during the diagnostic process, dental X-ray imaging has become the cornerstone for dental practitioners worldwide^8,9^. Radiography plays a vital function in supporting dentists’ imaging assessment, helping them provide comprehensive clinical diagnoses and preventive inspections of tooth structures^6^.

**Figure 1:**
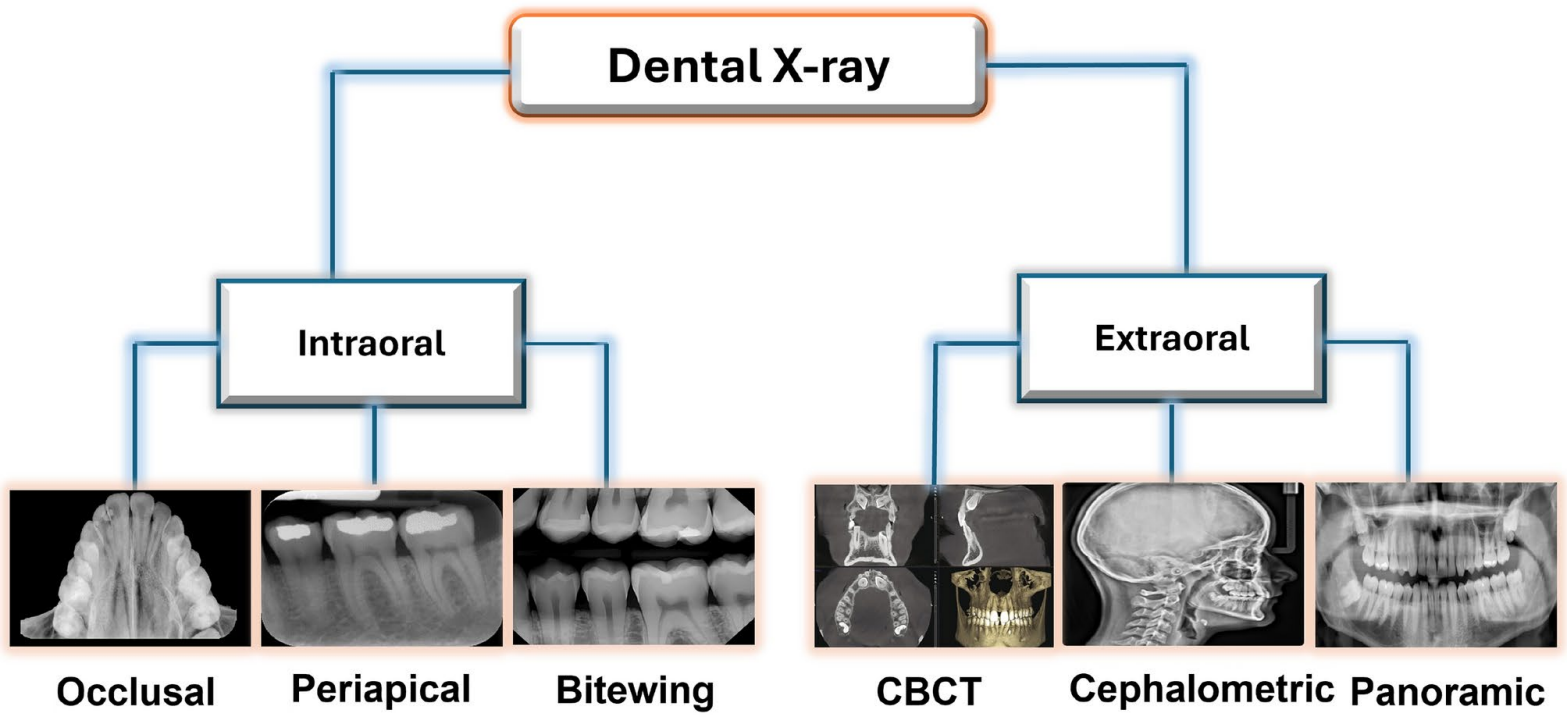
Different examples of the dental X-ray images.

In dentistry, X-rays are divided into two categories to record slightly different views of the mouth^10,11^: intraoral, where the X-ray image is obtained inside the mouth (i.e., bitewing, periapical, and occlusal X-rays), and extraoral, where the X-ray image is obtained outside the patient’s mouth (i.e., panoramic X-rays, Cephalometric projections, computed tomography (CT)). Figure 1 shows different examples of the dental X-rays images.

Analysis of dental X-rays can be a tedious treasure hunt, even for the most skilled dentists. Due to their diverse forms and sizes, teeth can readily conceal cavities and other indentation issues. Due to this, sorting through mountains of X-rays by hand is slow and prone to error, leaving important details out frequently^12^. Digital tools, such as a reliable metal detector, can be helpful in this situation. In order to help researchers and dentists identify hidden dental problems much more quickly and efficiently, automating this process with computer programs could be revolutionary^13–15^.

Medical diagnostics have seen a revolution in the last few decades, with machine learning (ML) and artificial intelligence (AI) taking the lead, particularly in the analysis of medical images. Dentistry has not been excluded from this innovative wave^16–25^. The expanding wealth of dental X-rays and scans has spurred the development of ML, specifically for oral and dental imaging. Convolutional neural networks (CNNs) are becoming a popular tool in this field because of their remarkable capacity to learn from intricate, hidden patterns. They are perfect for identifying dental issues that might otherwise go undetected because of their quick development and skill at identifying important details from blurry medical images^26^.

Inspired by the unrealized potential of unexplored feature extraction techniques in dental image analysis, this work explores methods that have not yet been applied to this research area. With proper application, these techniques could potentially reveal incredibly instructive information from dental X-rays. In order to overcome the current limitations of dental image diagnosis and achieve a more comprehensive picture, we apply a fusion approach that combines the strengths of these diverse techniques.

In this study, we propose a hybrid framework that combines MobileNetV2 and Swin Transformer, leveraging their distinct and complementary strengths to optimize feature extraction for dental disease diagnosis via X-ray imaging. MobileNetV2 was chosen for its efficient and lightweight architecture, which is especially useful in clinical settings where computational resources may be limited. Its design, which uses depthwise separable convolutions, significantly reduces the number of parameters and computational costs while retaining high performance in capturing fine-grained local features. This is especially important for dental X-ray images, where minute details such as small cavities or early signs of periodontal disease must be accurately detected to provide timely and effective care.

Furthermore, MobileNetV2’s efficient operation on mobile devices and embedded systems paves the way for point-of-care diagnostics, allowing for real-time analysis and decision-making in various healthcare settings. The model’s ability to handle variations in scale and position within images contributes to its effectiveness in processing a wide range of dental X-ray images, including those with varying resolution and quality.

On the other hand, Swin Transformer has been included because of its advanced ability to model long-range dependencies and capture global contextual information via a hierarchical self-attention mechanism. Unlike traditional CNNs, which are limited by their local receptive fields, the Swin Transformer captures complex and non-local interactions throughout the image. This is especially useful in dental disease diagnosis, where structural patterns and relationships between different parts of the image can reveal critical information about conditions like bone loss, infection spread, and tooth alignment.

Furthermore, the Swin Transformer’s ability to process images at multiple scales using its shifting window technique enables it to maintain a high level of detail while comprehending larger image contexts. This is critical for accurately diagnosing diseases that appear on different spatial scales within X-ray images, such as detecting caries progression or assessing the overall health of the jawbone. By integrating MobileNetV2 and Swin Transformer in a dual-stream approach, our framework capitalizes on the strengths of both architectures: MobileNetV2’s efficiency and local feature extraction capabilities and Swin Transformer’s prowess in capturing global context and long-range dependencies.

This synergistic combination enhances the model’s ability to detect and classify dental diseases with high accuracy and ensures that the model is versatile and adaptable to various clinical scenarios. The dual-stream approach facilitates a comprehensive analysis of dental X-rays, allowing the model to understand the micro-level details and macro-level patterns essential for precise diagnosis. This integration ultimately results in a more robust and reliable diagnostic tool that can significantly improve the quality of dental care. Therefore, the main contributions of this work can be summarized as follows:

- **Enhanced Image Quality:** By addressing lighting variations and improving image contrast in dental radiographs, adaptive histogram equalization improves image quality and facilitates better feature extraction in later stages.
- **Enhanced Feature Representation:** To produce a thorough representation of dental anomalies, features extracted by MobileNetV2 (effective local features) and Swin Transformer (strong global context capture) are combined.
- **Enhancing Performance:** Based on benchmark dataset evaluation metrics, such as accuracy, specificity, sensitivity, Dice coefficient, and Matthews correlation coefficient, the proposed system achieves better performance than current dental disease diagnosis methods.
- **Computational Efficiency:** Effective local feature extraction is achieved using MobileNetV2, a lightweight CNN, which may enable deployment in resource-constrained environments.
- **Robust Classification:** Increases generalizability and robustness of the classification process by using a bagging ensemble classifier, the proposed system results in more accurate predictions.

The rest of this research study is organized as follows. Section 2 discusses the previous studies of dental diseases using medical image analysis using two categories of feature extraction: handcrafted and deep learning feature-based techniques. Section 3 presents a detailed explanation of the proposed framework, which consists of five stages: preprocessing, feature extraction, feature normalization, feature fusion, and classification. Section 4 presents the experimental results, starting with the description of the utilized datasets and evaluation metrics, as well as the obtained results and its discussion. Finally, the conclusion and future work are presented in Sect. 5.

## Related work

Dental image analysis is an area of research that is expanding quickly and has been the subject of many publications. In the following subsections, the current approaches for extracting discriminating features from dental images will be reviewed. These techniques can be divided into handcrafted feature-based methods and deep learning feature-based methods.

### Handcrafted feature-based methods

Although medical image analysis has seen considerable success with deep learning models, many computer vision tasks benefit significantly from handcrafted feature extraction. This subsection examines previous research that uses manually created features to classify dental diseases from radiographs. These studies give insights into the underlying features of images that are important for dental diagnosis.

Geetha and Aprameya^27^ proposed a method based on texture features for dental caries diagnosis in digital image radiographs. First, the Laplacian filter was applied for object sharpening, and morphological filters and adaptive thresholds were used for segmentation. Second, grey level co-occurrence matrix (GLCM)^28–30^ and grey level difference method (GLDM)^31,32^ were used to extract texture features. Finally, a support vector machine (SVM) was used for the classification process. This system classified images into normal and caries. This system achieved 96.8%, 86.6%, and 96.1% for accuracy, sensitivity, and specificity, respectively.

Geetha and Aprameya^33^ presented a method for X-ray dental caries image classification. This method depended on a Gaussian low pass filter applied in the frequency domain for feature extraction. Finally, SVM was used for the classification step. There were 105 images of caries and normal cases. The dataset was divided into 10-fold cross-validation. SVM classifier achieved 97.3% accuracy. Jusman et al.^34^ proposed a method for dental caries classification. Firstly, image preprocessing was applied to these images. Secondly, GLCM was used for texture feature extraction. Finally, SVM and K-nearest neighbors (KNN) were used for classification. The number of images was 396. The SVM achieved 83.3% for accuracy and 91.4% for the KNN classifier.

Singh et al.^35^ proposed a method for classifying images into caries and normal images. Some texture feature techniques were used for feature extraction, such as GLCM, local binary pattern (LBP), local binary gray level co-occurrence matrix (LBGLCM), and gray level run length matrix (GLRLM). After that, principal component analysis (PCA) was applied for feature selection. Finally, the AdaBoost classifier was used and achieved an accuracy of 99.7%, 98.7%, 97.9%, and 90.8% for LBGLCM, GLCM, GLRLM, and LBP.

Yaduvanshi et al.^36^ proposed an ML method for oral cancer classification. This method depended on a modified local binary pattern (MLBP) for extracting texture features from images. This modified algorithm presented the connectivity in local and global image regions. KNN, decision tree (DT), and SVM classifiers were applied for the classification process. SVM achieved an average accuracy of 91.3% and 94.4% for 100x and 400x resolution images, respectively.

### Deep learning feature-based methods

Deep learning (DL) is advancing so quickly that researchers are looking into how it might be used for automated dental diagnosis. This subsection explores current DL methods used for dental radiography analysis. In order to increase dentists’ diagnostic efficiency and accuracy, we will examine how these studies use DL architectures to extract features and carry out classification tasks. For example, Prajapati et al.^37^ proposed a dental disease classification method using CNNs with transfer learning. They worked on classifying three popular diseases: dental caries, periapical infection, and periodontitis. A transfer learning with a VGG16 pre-trained model achieved 88.4%

Singh and Sehgal^38^ developed automatic dental image classification architecture for classifying dental caries into six G.V. black classes using an optimal CNN-LSTM classifier with dragonfly optimization. They achieved an accuracy of 96.0% on a dataset consisting of dental X-ray periapical 1500 images collected from two dental clinics in New Delhi, India. Megalan et al.^39^ introduced a system for detecting cavities in dental X-rays. This system used the GoogleNet Inception v3 architecture to implement CNNs, a deep learning algorithm. To train the network, they assembled a dataset of 480 bite-wing x-rays from the Elsevier database. A noise-reduction filter and standard format resizing were used as preprocessing techniques for the images. Cavities were detected with an accuracy of 86.7% by the trained network.

Lian et al.^40^ looked into using DL to identify and categorize the severity of cavities in panoramic dental X-rays. A reference standard of cavity locations and depths was developed by dentists through a thorough analysis of a large dataset consisting of over 1160 dental panoramic films. DenseNet121 was trained to classify cavity depth (affecting outer, middle, or inner dentin), and nnU-Net was trained to detect cavities. With a 98% accuracy rate, the nnU-Net model demonstrated remarkable performance in cavity detection. Moreover, DenseNet121 achieved over 95% overall accuracy for specific depths.

Using panoramic radiographs, Vinayahalingam et al.^41^ investigated the DL method to detect cavities in third molars. Using a dataset of 400 labeled images showing cavities in these particular teeth. They trained a MobileNet V2. After training, the model was evaluated on an additional 100 images. The system classified the presence or absence of cavities with 87% accuracy. Hasnain et al.^42^ proposed a method for dental disease diagnosis and classification from X-ray images. The dataset consisted of 126 images with labels as affected or normal. Firstly, data augmentation was applied to increase the size of the dataset. A CNN model consisted of convolutional, max-pooling, flattened, dense, and output layers. This method achieved 97.8% accuracy.

Kadarina et al.^43^ proposed a dental caries classification and diagnosis method based on depthwise separable convolutional (DSCon). The reduction of trainable parameters in DSCon successfully reduces the computational cost of conventional CNNs. As a result, the DSCon model is reduced by 91.4% compared to the conventional CNN model. Park et al.^44^ proposed a method for classification and clustering dental implant diseases. Firstly, it depended on the VGG16 DL model and justed its parameters for classification. Secondly, the clustering method used k-means, which segmented dental implant regions. Table 1 indicated comparing the latest dental disease diagnosis and classification research.

There are various studies for dental disease diagnosis and classification. Some of them depended on handcrafted methods, and others on DL methods. The reviewed studies have limitations that limit their practical applicability for diagnosing dental diseases. First, insufficient training data concentrating on particular anomalies leads to models that exhibit generalizability difficulties. Second, some feature extraction methods may be less successful in capturing important details when there is low image quality due to noise and artifacts. Finally, some methods have trouble distinguishing between dental abnormalities that overlap or have similar visual characteristics, which could result in incorrect classifications in complicated cases.

Our suggested deep learning framework addresses the constraints in dental diagnosis related to data, image quality, and feature extraction. We use a lightweight architecture (MobileNetV2) for effective training in order to address the lack of data. Image quality is improved for feature extraction through preprocessing methods like equalization. Furthermore, to capture robust features even from low-quality images and effectively distinguish between complex or overlapping dental abnormalities, the model combines MobileNetV2 for local details and Swin Transformer for global context. This thorough method opens the door to more reliable and accurate dental disease diagnosis by addressing essential shortcomings.

**Table 1:** The comparison of some current related studies.

Paper	Features extraction	Classification	Dataset	Disease	Accuracy (%)	Limitations
Jusman et al.^34^	GLCM	SVM and KNN	Private DS	Caries	83.3% for SVM 91.4% for KNN	Low contrast images
Singh and Sehgal^38^	CNNLSTM	Softmax	1500 images	Caries	96.0%	
Singh et al.^35^	LBGLCM, GLCM, GLRLM, and LBP	AdaBoost	Caries	Private DS	99.7% for LBGLCM, 98.7% for GLCM, 97.9% for GLRLM, and 90.8% for LBP	No preprocessing applied
Geetha and Aprameya^33^	Gaussian filter	SVM	Caries	Private DS	0.973%	Small Dataset
Yaduvanshi et al.^36^	MLBP	SVM	Oral cancer	Mendeley dataset^45^	91.36% for 100x 94.44% for 400x	Low contrast images
Geetha and Aprameya^27^	GLCM and GLDM	SVM	Private DS from India	Caries	96.88%	Small dataset
Prajapati et al.^37^	VGG16	Softmax	RVG	caries periapical infection periodontitis	88.46%	Small dataset
Megalan et al.^39^	GoogleNet Inceptionv3	Softmax	Bitwings from the Elsevier database	caries	86.7%	Small dataset & low image quality
Vinayahalingam et al.^41^	MobileNet V2	Softmax	private DS from the Department of Oral & Maxillofacial Surgery of Radboud University Nijmegen Medical Centre	caries	87.0%	Focus only on third molars
Lian et al.^40^	UNet & DensNet121	Softmax	private dataset	caries	98% for UNet and 95% for DensNet121	Overlapping anatomical structures
Hasnain et al.^42^	CNN	Dense	Private DS	Normal & Affected	97.87%	Small dataset & low image quality
Kadarina et al.^43^	DSCNN	Dense	Private DS	Caries	91.49%	Small dataset
Park et al.^44^	VGG16	Dense	Private DS	implanted	99.4%	Low quality images

## Proposed framework

**Figure 2:**
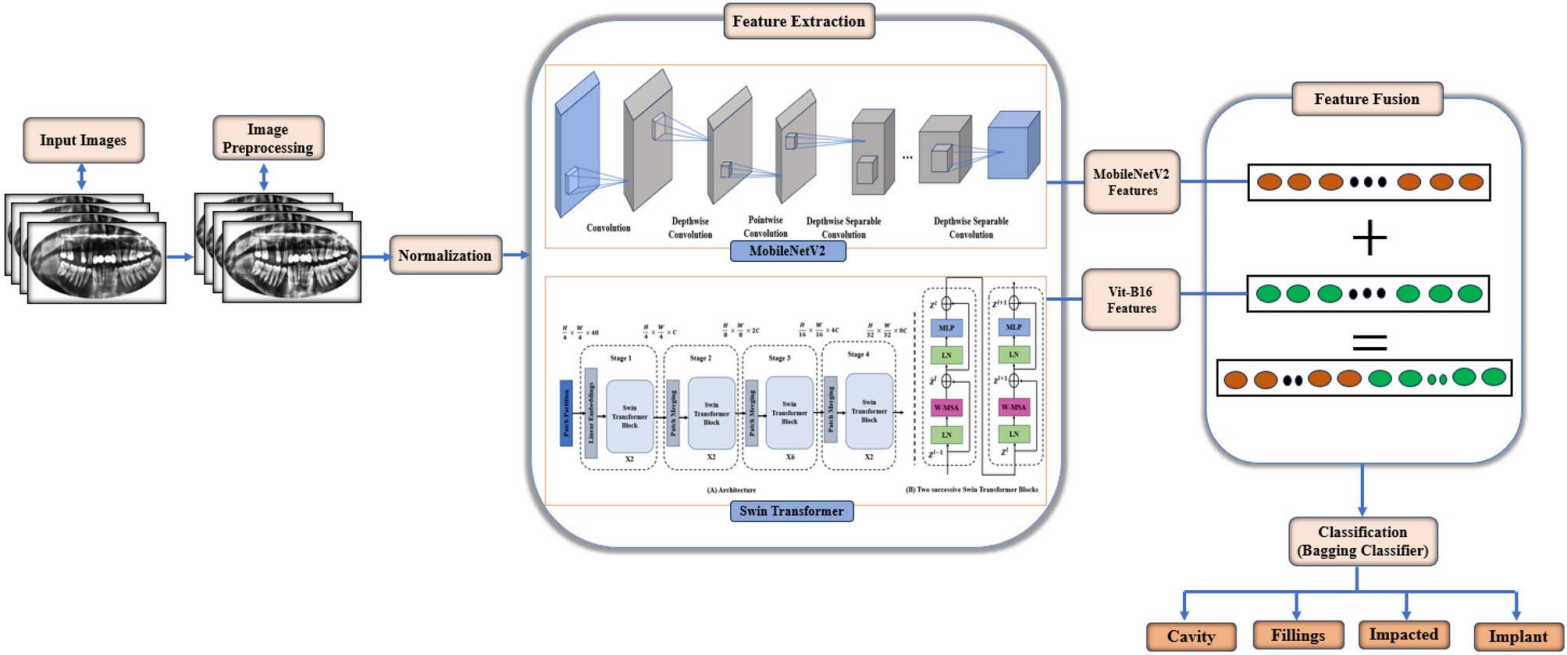
The dental diseases diagnosis based on MobileNetV2 and swin transformer framework.

This section describes the proposed DL framework for diagnosing dental diseases in detail. With a combination of approaches, our suggested model addresses the shortcomings found in current approaches. We use MobileNetV2, a lightweight and effective CNN architecture, to extract local features from dental radiographs. A Swin Transformer is also incorporated to capture long-range dependencies and global context within the images. This fusion of local and global features intends strong and thorough representations of dental anomalies. A preprocessing step is also included in the framework to improve image quality. We use a bagging ensemble classifier to achieve robust and generalizable classification performance by utilizing multiple instances of the feature extraction architecture trained on bootstrap samples of the training data. The architecture of our suggested method for diagnosing dental diseases is shown in Fig. 2.

### Image preprocessing

Image processing is a computer-based procedure that changes recorded electronic (digital) images to enhance their quality to be suitable for the feature extraction stage^46,47^. Two preprocessing methods have been applied (i.e., image standardization and adaptive histogram equalization), which will be discussed in the following subsections.

#### Adaptive histogram equalization

Image quality enhancement is critical in medical image analysis^48,49^. Dental radiographs are an essential part of the diagnostic process. On the other hand, inconsistent lighting during the picture capture process may result in blurry images and low contrast. This may make subtle details more challenging to see, which is essential for correctly diagnosing dental diseases. The suggested framework uses the preprocessing method of adaptive histogram equalization (AHE) to overcome this difficulty. This method achieves higher results in image quality enhancement^50,51^. AHE seeks to improve image contrast by enhancing pixel intensity distribution within specific regions of the dental radiograph.

Traditional histogram equalization aims to achieve a uniform distribution by redistributing pixel intensities, represented as p(i), throughout the entire image. This can be expressed in Eq. (1):

1 $$\begin{aligned} p'(i) = \frac{M - 1}{N} \sum _{j=0}^{i} p(j) \end{aligned}$$

where *M* is the total number of intensity levels, typically 256 in grayscale pictures. The total number of pixels in the picture is *N*. After equalization, the new probability of intensity level *i* is denoted by $p'(i)$.

There may be restrictions to this method, especially when it comes to dental radiographs with various anatomical structures. To overcome this, AHE applies histogram equalization to smaller image subregions (windows) of size $w \times w$. The AHE mechanism is broken down as follows:

- **Divide the Image:** The image is separated into subregions (*W*) that are either overlapping or not.
- **Compute Local Histogram:** A local histogram, $h_k(i)$, is computed for every window $w_k$, where *k* is the window index. This histogram illustrates the distribution of pixel intensities in that particular area.
- **Histogram Stretching:** This technique modifies the local histogram to produce a more even distribution. Techniques like clipping or transformation of the probability distribution function may be used. One popular method is to clip the histogram to reduce the impact of extremely high or low-intensity pixels. Assign $Clip_L$ to the lower clipping limit and $Clip_H$ to the upper clipping limit. Next, the clipped local histogram $h'_k(i)$ is computed as follows in Eq. (2):
  2 $$\begin{aligned} h'_k(i) = {\left\{ \begin{array}{ll} h_k(i), & \text {if } Clip_L \le h_k(i) \le Clip_H \\ 0, & \text {if } h_k(i) < Clip_L \\ 1, & \text {if } h_k(i) > Clip_H \end{array}\right. } \end{aligned}$$
- **Probability Distribution Function Transformation:** An alternative method to obtain a desired distribution shape (e.g., closer to a uniform distribution) is to transform the local histogram using a function. This frequently entails transforming the cumulative distribution function (CDF). The clipped local histogram is used to compute the new probability distribution function (PDF), $p'_k(i)$ (Eq. 3):
  3 $$\begin{aligned} p'_k(i) = \sum _{j=0}^{i} h'_k(j) \end{aligned}$$
- **Window Replacement:** Depending on the method selected, the new intensity values obtained from the processed local histogram $(h'_k(i)$ or $p'_k(i))$ are used to replace the original pixel intensities within the window $w_k$.
- **Merge Windows:** Combining the processed windows recreates the entire image with improved contrast in specific areas.

**Figure 3:**
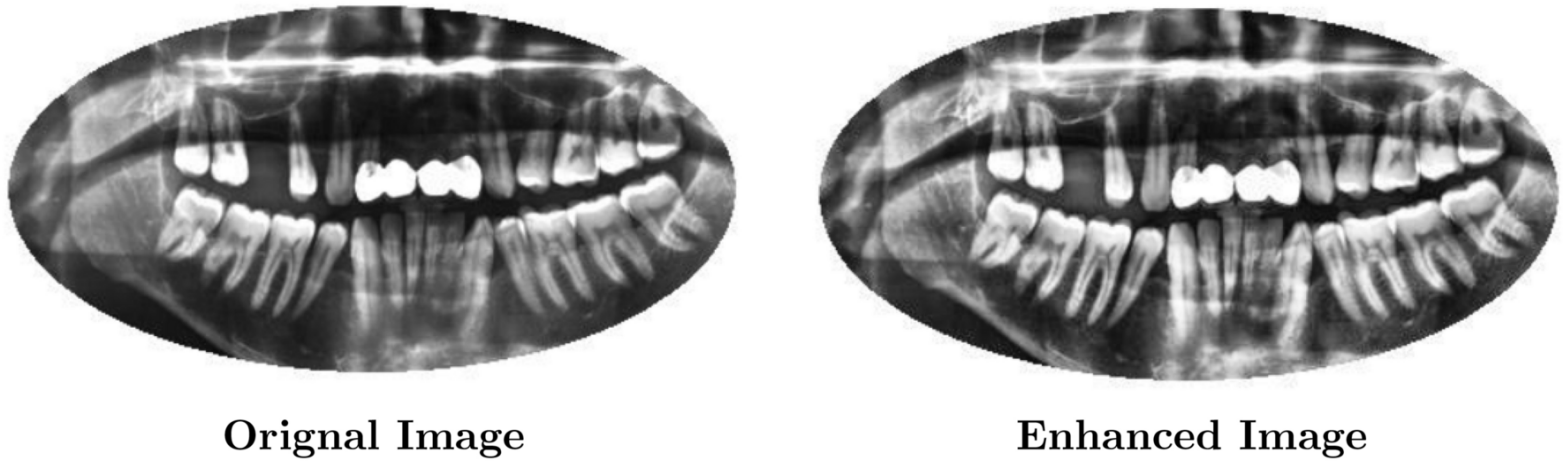
The AHE technique for dental image enhancement.

AHE is a useful preprocessing technique in the proposed framework for dental radiographs. It adjusts the local contrast based on mathematical transformations of the pixel intensity distribution to highlight the details essential for diagnosing dental diseases. AHE improves contrast in particular image areas without over-amplifying noise in other areas. Figure 3 indicates an example of images after applying this technique.

#### Image standardization

The preprocessing step seeks to enhance the quality of the images to help in the following stages. Using the image exactly as it is and passing it through a deep neural network could make computing huge numbers more difficult. Hence, in this paper, normalized images are resulted by subtracting the mean pixel values from their pixel values and then dividing them by the standard deviation of the values of pixels as in Eq. (4).

4 $$\begin{aligned} I_{norm}=\frac{I(x,y)-I_{mean}}{I_{sdv}} \end{aligned}$$

where $(I_{norm}$ is the normalized image while $I_{mean}$ and $I_{sdv}$ are the image mean and standard deviation, respectively.

Each pixel intensity in the dental radiography images was first scaled to the [0, 1] range by dividing by 255. This scaling ensures that all input features contribute equally to the learning process. We subtract the mean values [0.485, 0.456, 0.406] and divide by the standard deviations [0.229, 0.224, 0.225] for each RGB channel. This step ensures that the input distribution closely matches the distribution the models encountered during their initial training, allowing for more effective feature extraction and transfer learning.

#### Resizing

Resizing the images to the proper dimensions is critical for ensuring compatibility with the pre-trained models’ input specifications. MobileNetV2 and Swin Transformer require input images of specific sizes to function properly. In our study, we did the following resizing steps:

- Image Dimensions: Each dental radiography image was resized to 224x224 pixels. This size was chosen to match the input dimensions required by MobileNetV2 and is widely used in computer vision tasks. Consistent image dimensions are critical for batch processing because they ensure the models can handle inputs without encountering dimensionality mismatches.
- Interpolation Method: Bilinear interpolation was used during the resizing process to preserve the spatial integrity of the dental structures depicted. This technique aids in maintaining significant features and information necessary for a precise diagnosis.

By carefully following these preprocessing steps, we ensured the dental radiography images were formatted adequately for input into the pre-trained MobileNetV2 and Swin Transformer models. These preprocessing techniques not only help with feature extraction and model training but they also improve the overall performance and reliability of our hybrid dental disease detection system.

### Feature extraction

In this stage, discriminative information is found and extracted to distinguish between various dental diseases^52–55^. This is a vital stage where the classification performance is significantly affected. A detailed description of the utilized feature extractors is presented next.

#### MobileNetV2

**Figure 4:**
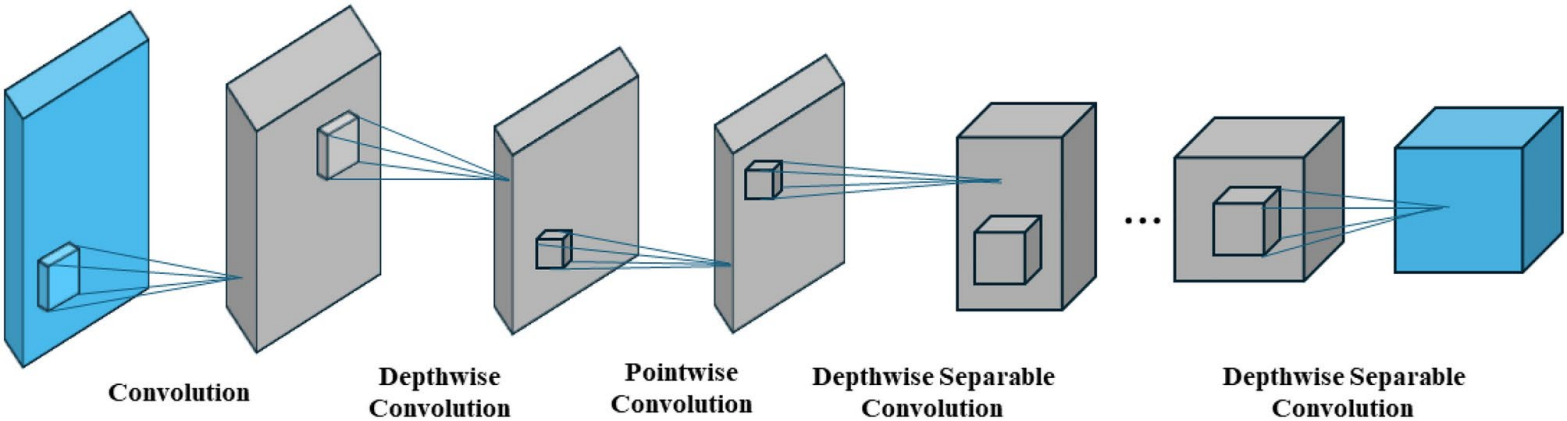
The MobileNet architecture.

Accurate diagnosis of the disease depends on extracting informative features from dental radiographs. For this crucial task, our suggested framework uses MobileNetV2, a CNN architecture that is both lightweight and effective. The implemented model utilizes the MobileNet architecture as a base, pre-trained on the ImageNet dataset, for feature extraction, as shown in Fig. 4. For dental radiograph analysis, MobileNetV2 has the following benefits:

- **Computational Efficiency:** Clinical settings with limited resources are frequently the need for dental diagnostic system deployment. Comparing MobileNetV2 to conventional CNN architectures, comparatively fewer parameters are needed to achieve high accuracy. It is, therefore, appropriate for real-world applications due to its quicker training times and reduced computational requirements.
- **Emphasis on Local Features:** MobileNetV2 performs exceptionally well in identifying local spatial features in images, which are crucial for differentiating between different patterns and textures linked to different dental abnormalities in radiographs.

To accomplish effective feature extraction, MobileNetV2 combines several depthwise separable convolution blocks. There are two primary parts to these blocks:

- **Depthwise Convolution:** To extract local spatial information from each input channel, this layer applies a filter. The depthwise convolution operation with a filter *F* of size $k \times k$ can be mathematically represented as follows in Eq. (5) for an input feature map *X* of size $H \times W \times C$ (where *H* and *W* are the height and width and *C* is the number of channels):
  5 $$\begin{aligned} Y^{dw}(i, j, c) = \sum _{m=1}^{k} \sum _{n=1}^{k} X(i + m - 1, j + n - 1, c) \cdot F(m, n, c) \end{aligned}$$
  where the output at position (*i*, *j*) in the c-th channel of the feature map following depthwise convolution is represented as $Y^{dw}(i, j, c)$. The filter size $k \times k$ is iterated over by the summation.
- **Pointwise Convolution** ($1 \times 1$**convolution**): To increase model efficiency, this layer uses a $1 \times 1$ convolution filter to decrease the number of channels in the output feature map. The pointwise convolution operation with a filter $F_p$ of size $1 \times 1 \times C_out$ (where $C_out$ is the number of output channels) for an input feature map $Y^{dw}$ of size $H \times W \times C_in$ (where $C_in$ is the number of channels after depthwise convolution) can be expressed as follows (Eq. 6):
  6 $$\begin{aligned} Y(i, j, c_out) = \sum _{c=1}^{C_{in}} Y^{dw}(i, j, c) \cdot F_p(1, 1, c, c_out) \end{aligned}$$

where the output at position (*i*, *j*) in the feature map’s $c_out-th$ channel, following pointwise convolution, is represented by the symbol $Y(i, j, c_out)$. The input channels $C_{in}$ are iterated through by the summation.

Achieving a good balance between computational efficiency and feature extraction capability, MobileNetV2 combines pointwise convolutions with depthwise separable convolutions. MobileNetV2 introduces linear bottlenecks into the depthwise separable convolution blocks, improving efficiency. Reduced channel counts within the feature map before pointwise convolution cause these bottlenecks. The two main goals of this strategy are to:

- **Lower Computational Cost:** The total computational load is decreased by lowering the number of channels that the pointwise convolution processes. This results in less memory requirements and quicker training.
- **Encourage Feature Compression:** With fewer channels available, the bottleneck compels the model to learn more informative representations. Working with small training datasets, as frequently in medical imaging applications, may result in better generalization performance and avoid overfitting.

The linear bottleneck can be expressed mathematically as (Eqs. 7, 8):

7 $$\begin{aligned} Z(i, j, c)= & ReLU(W_1 \cdot Y^{dw}(i, j,.) + b_1) \end{aligned}$$

8 $$\begin{aligned} Y(i, j, c_out)= & W_2 \cdot Z(i, j,.) + b_2 \end{aligned}$$

Within a few of its depthwise separable convolution blocks, MobileNetV2 additionally includes residual connections. By adding the original input feature map to the block’s output, these connections enable the model to learn the identity mapping. In deeper networks, in particular, this can help mitigate the vanishing gradient issue during training and possibly enhance the model’s capacity to pick up on intricate details in dental radiographs. The residual connection can be represented as follows in Eq. (9):

9 $$\begin{aligned} Y(i, j, c_out) = F(X(i, j,.)) + Y^{dw}(i, j, c_out) \end{aligned}$$

where the non-linearity of the depthwise separable convolution block is represented by *F*. The combination of linear bottlenecks and residual connections allows MobileNetV2 to learn complex representations for precise dental disease classification while maintaining a good balance between efficiency and feature compression. MobileNetV2 is a useful option for feature extraction in our dental radiograph analysis framework because of its effective architecture, which includes depthwise separable convolutions, linear bottlenecks, and residual connections. Its ability to preserve computational efficiency while capturing informative local features makes real-world applications in clinical settings possible.

#### Swin transformer

**Figure 5:**
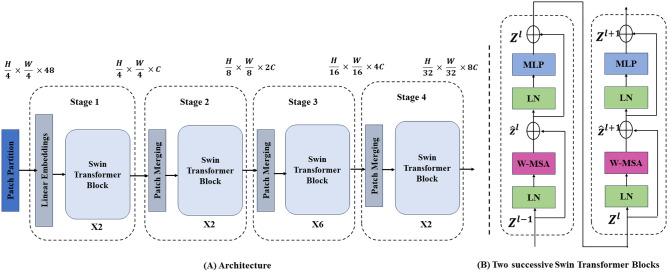
The Swin Transformer architecture.

Using the Swin Transformer architecture, our suggested framework can extract global context and long-range dependencies from dental radiographs. Swin Transformers are superior to traditional CNNs at capturing relationships between distant image regions, which can be important for accurately diagnosing dental diseases. Traditional CNNs mainly focus on local spatial features. As shown in Fig. 5, the Swin Transformer architecture is constructed from a number of essential elements.

Partitioning a dental radiograph into smaller image patches is the first step in the process. This enables the model to learn relationships between data points by processing the data in smaller chunks. Patch size is a hyperparameter that can be adjusted to strike a compromise between the capacity to capture spatial information and computational efficiency. The next step is to embed each patch using a linear projection layer into a higher-dimensional vector space. The raw pixel data is changed via this embedding process into a representation better suited for the transformer architecture. The patch embedding has the following mathematical representation (Eq. 10):

10 $$\begin{aligned} Z_j = W_e \cdot X_j + b_e \end{aligned}$$

where Patch *j* is represented embeddedly by $Z_j$. Patch *j* is represented by $X_j$ in its original pixel representation. The weight matrix for embedding is denoted by $W_e$. The embedding bias vector is represented by $b_e$.

The hierarchical architecture of the Swin Transformer’s stage transformer blocks forms its foundation. In each step, a feed-forward network comes after several self-attention layers. The self-attention layer determines the attention scores that separate each patch in a local window from every other patch. This allows the model to capture contextual information and long-range dependencies. These scores indicate how vital each patch is in relation to the current patch. The self-attention mechanism can be expressed as the following:

11 $$\begin{aligned} Attention(Q, K, V) = softmax\left( \frac{Q \cdot K^T}{\sqrt{d}}\right) \cdot V \end{aligned}$$

where the embedded patch representations are linearly projected to produce the query, key, and value matrices, respectively, *Q*, *K*, and *V*. The embedded patch representation’s dimension is denoted by *d*. The attention scores are normalized to a probability distribution by softmax. The feed-forward network adds non-linearity to the model, enabling it to pick up increasingly intricate relationships between patches. In most cases, two fully linked layers with a non-linear activation function (such as ReLU) make up the feed-forward network.

Swin Transformer’s use of the shift window multi-head self-attention (SW-MSA) in the self-attention layer is a significant innovation. In contrast to conventional self-attention, which is restricted to a local window, SW-MSA enables the model to focus on informative regions that extend outside of the local window. By doing this, the model can gradually capture long-range dependencies throughout the entire image by adjusting the window for each layer within a stage. The SW-MSA mechanism is represented using the following equation:

12 $$\begin{aligned} Attention_{SW-MSA}(Z_j) = \sum _{w \in {\mathcal {W}}} \frac{1}{|{\mathcal {W}}|} \cdot Attention(Q_j^w, K_j^w, V_j^w), \end{aligned}$$

where ${\mathcal {W}}$ is the set of shifted windows for the current layer, and $Z_j$ is the embedded representation of patch *j*. The query, key, and value matrices for patch *j* inside window *w* are $Q_j^w, K_j^w, and V_j^w$. The summation goes through each window in the set ${\mathcal {W}}$ iteratively.

There are various benefits to the Swin Transformer architecture for dental radiograph analysis. Swin Transformers are excellent at learning long-range dependencies through the self-attention mechanism, in contrast to CNNs, which find it challenging to discern relationships between far-off image regions. In diagnosing dental diseases, this is especially crucial because minute differences in the texture and pattern of the radiograph in different areas can point to particular abnormalities.

Swin Transformer blocks’ hierarchical architecture enables the model to learn features at various scales. Later stages can learn more about global features and the contextual relationships among the local details captured in the initial stages. This multi-scale feature representation is essential for accurately classifying dental diseases. Swin Transformers are an attractive option for dental radiograph analysis because of their adaptable patch size, scalable stage architecture, and effective feature learning mechanisms. With these features, we can customize the model to perform particular dental diagnostic tasks with computational efficiency.

#### Fine-tuning

In our proposed model, we used pre-trained versions of MobileNetV2 and Swin Transformer as backbone architectures for feature extraction. Leveraging pre-trained models provides the advantage of starting with weights that have already learned diverse features from large-scale datasets, such as ImageNet, resulting in better performance even with smaller labeled datasets specific to our task. We used fine-tuning to adapt these pre-trained models to our specific dental radiography dataset. Fine-tuning entails training the pre-trained model on our dataset again, but with certain changes to prevent overfitting:

1. Layer Freezing: We first froze the weights of the earlier layers in both MobileNetV2 and Swin Transformer. These layers typically capture low-level features such as edges and textures, widely applicable across image domains, including dental radiographs. By freezing these layers, we could preserve the learned features from the pre-training phase while reducing the risk of overfitting by preventing the model from over-adjusting to our specific dataset.
2. Gradual Unfreezing and Fine-Tuning: After training the higher layers, which capture more task-specific features, we gradually unfroze some of the model’s lower layers. This method enabled us to fine-tune the model gradually, beginning with the layers closest to the output, which are more task-specific, and gradually adapting the more generic layers.
3. Regularization Techniques: In addition to fine-tuning, we used dropout layers within the final classification layers, particularly in fully connected layers, to prevent neuron co-adaptation and reduce overfitting. During training, the dropout layers randomly deactivate a subset of the neurons, forcing the model to rely on a broader set of features, thus improving its robustness.
4. Reduced Learning Rate: In the fine-tuning phase, we used a reduced learning rate to make smaller updates to the model weights. This careful adjustment aids in fine-tuning the pre-trained model without excessively adjusting the learned weights, thereby preventing overfitting. A lower learning rate ensures that the model improves its feature extraction capabilities without deviating too far from the useful patterns discovered during the initial pre-training.

### Feature fusion

Feature-level fusion methods work with data at higher processing levels than pixel-level methods do^56,57^. The first thing that is usually employed is feature extraction methods. After that, the fusion process is carried out by concatenating several extracted feature pointsets, as shown in Eq. (13).

13 $$\begin{aligned} F_{final}=[F_{MN} + F_{ST}] \end{aligned}$$

where $F_{final}$ is the final concatenated feature vector, $F_{MN}$ is the MobileNetV2 extracted feature vector, $F_{ST}$ is the Swin Transformer extracted feature vector, the plus (+) sign denotes that concatenation operation.

The proposed model employs a dual-stream architecture that combines MobileNetV2 and Swin Transformer to improve feature extraction for diagnosing dental conditions such as fillings, implants, impacted teeth, and cavities. In this method, input dental X-ray images are processed in two parallel streams, each utilizing the strengths of one of the architectures.

For dental radiograph analysis, our suggested framework combines the complementary advantages of MobileNetV2 and Swin Transformer via feature fusion. By combining features taken from various modalities or network architectures, feature fusion seeks to produce a richer and more insightful feature representation. In our scenario, we integrate the global context and long-range dependencies that the Swin Transformer has learned with the local detail-oriented features that MobileNetV2 has captured.

For dental radiograph analysis, our suggested framework combines the complementary advantages of MobileNetV2 and Swin Transformer by utilizing late fusion in conjunction with element-wise summation. Combining element-wise summation with late fusion offers a potent and adaptable way to utilize MobileNetV2 and Swin Transformer’s complementary advantages. This fusion strategy can significantly improve the accuracy and generalizability of dental disease classification within our proposed framework by carefully addressing dimensionality mismatches and investigating interpretability aspects.

The MobileNetV2 stream is primarily responsible for capturing detailed local features using depthwise separable convolutions. This enables the model to detect and differentiate fine-grained details in X-ray images, such as filling contours, implant integration with surrounding bone, and cavity locations. MobileNetV2’s ability to focus on these micro-level details is critical for accurately diagnosing conditions where minor but significant variations in the image indicate dental health issues.

In parallel, the Swin Transformer stream captures global contextual features from the same input images. The Swin Transformer’s hierarchical self-attention mechanism excels at detecting long-range dependencies and broader spatial relationships within images. This capability is especially useful for diagnosing impacted teeth, where the spatial arrangement and relationship with neighboring teeth and bone structures are critical. Furthermore, the Swin Transformer’s ability to retain an understanding of the entire image context aids in determining the overall placement and condition of implants within the dental arch.

The features extracted by MobileNetV2 and Swin Transformer are then directly fused to form a unified feature representation that combines the benefits of local detail extraction and global context understanding. This fusion enables the model to analyze dental X-ray images comprehensively, ensuring that the fine details of dental structures and their broader anatomical context are considered in the diagnosis.

Integrating MobileNetV2 and Swin Transformer into a cohesive framework posed several challenges, particularly in balancing computational efficiency and ensuring effective feature integration. One major challenge was managing the computational load, as the Swin Transformer’s self-attention mechanism is more resource-intensive than MobileNetV2. To address this, we designed the architecture so that MobileNetV2 processes the initial layers of the image, reducing the input size and complexity before the features are passed to the Swin Transformer. This approach optimizes computational resources, allowing the Swin Transformer to operate more efficiently on a refined and relevant feature set.

### Classification

Once we have combined the representations of MobileNetV2 and Swin Transformer to extract informative features, the framework moves on to the classification phase. We use a robust and accurate dental disease classification from dental radiographs by combining an SVM as the base learner with a potent ensemble learning technique called bagging. Bagging helps prevent the overall model from overfitting to the particular training data by training multiple SVMs on different subsets of the data. This results in a more robust classifier that can more effectively generalize to unseen dental radiographs during prediction.

SVM is used as the base learner in the ensemble bagging classifier for each *N* partitions (or bags) through bootstrap sampling, with one partition used for each base learner produced by the SVM algorithm. After being trained by the corresponding data bag, this base learner enters a parallel process to generate the subsequent base learner. Voting for the classification from which the ensemble prediction is derived yields the final estimator. The class with the highest number of votes is designated as the final prediction by Eq. (14), which counts the number of votes each class receives from the base learners.

14 $$\begin{aligned} F(x) = \arg \max _y \left( \sum _{i=1}^{B} (f_i(x) == y) \right) \quad \end{aligned}$$

where *y* iterates over all possible class labels. The main benefit of using SVMs in our framework is their efficacious handling of high-dimensional feature spaces. The fused representation of Swin Transformer and MobileNetV2 features may be high-dimensional. Because SVMs are well-suited to handle high-dimensional data, they can efficiently learn the underlying decision boundaries for precise classification of dental diseases.

When analyzing dental radiographs, there are various benefits to using SVM and bagging together in the classification stage. When dealing with heterogeneous feature sets, such as those derived from the combination of MobileNetV2 (local features) and Swin Transformer (global features), bagging is especially useful. SVMs are renowned for their ability to withstand noise and data outliers. This is especially important when working with dental radiographs from real-world situations, which may have some noise or artifacts. The proposed framework aims to achieve accurate and dependable dental disease classification from dental radiographs by utilizing SVMs for robust classification with high-dimensional features and bagging for improved generalizability. When these methods are combined, they may provide a potent tool that helps dentists plan treatments and make early diagnoses.

The decision to use a bagging ensemble classifier in our model’s final classification stage was motivated by its ability to improve the predictability and generalizability of the results. Bagging, also known as Bootstrap Aggregating, creates multiple versions of a dataset using bootstrapping (random sampling with replacement) and then trains a base classifier on each version. The final prediction is made by averaging the outputs of these base classifiers, which reduces variance and helps to prevent overfitting.

Dental radiographs present a wide range of variability in disease features, such as the size and shape of cavities, the positioning of implants, and the orientation of impacted teeth. This variability can make it challenging for a single classifier to generalize well across different cases. Bagging addresses this issue by creating multiple versions of the training dataset through bootstrapping, leading to training multiple base classifiers. The aggregated predictions from these classifiers help capture the data’s diverse patterns, making the model more robust to variations and reducing the risk of overfitting to specific patterns.

## Experimental results

### Datasets details

**Figure 6:**
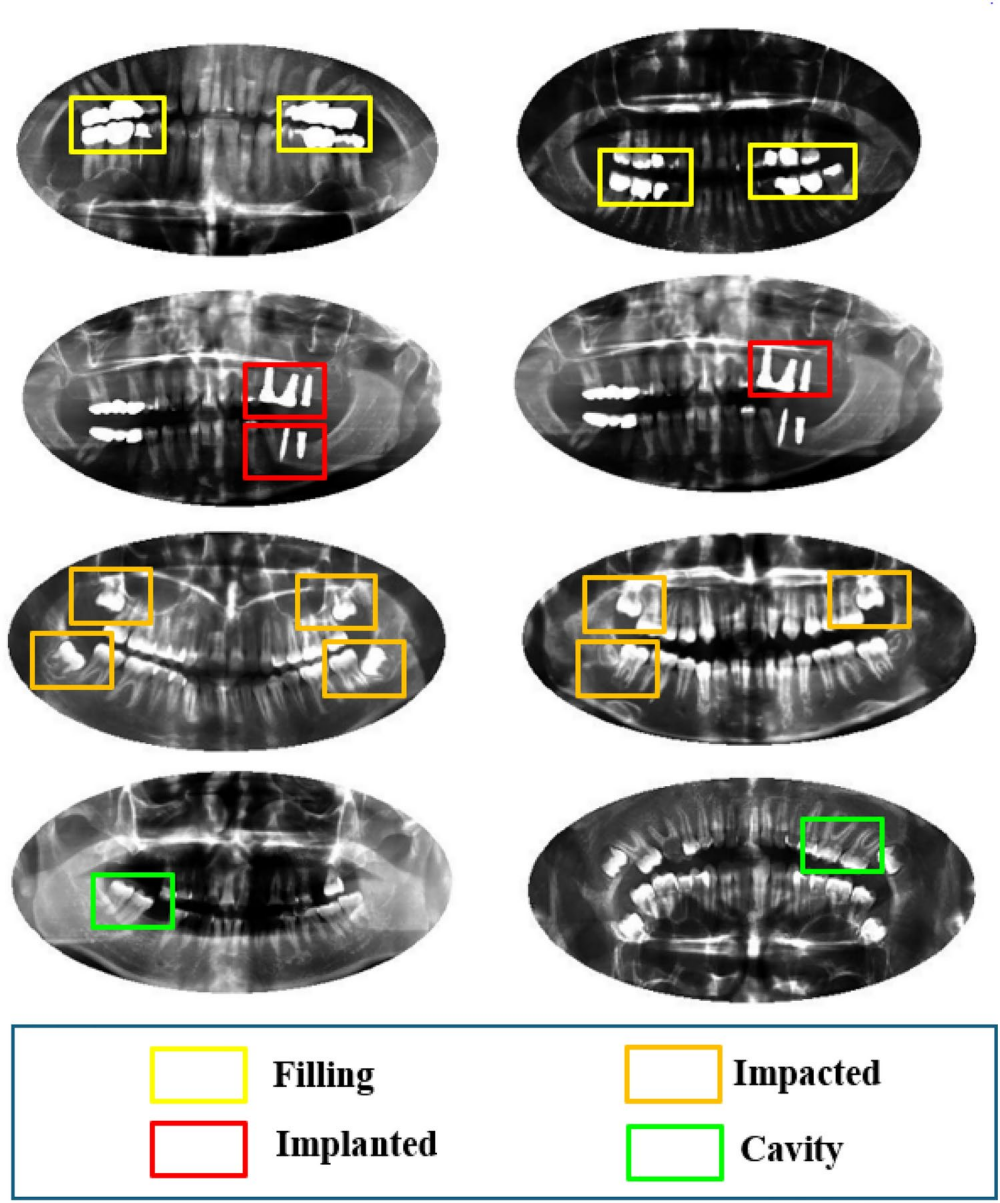
Some examples from the used dataset images with four labels (filling, implanted, impacted, and cavity).

The proposed framework was evaluated on dental radiography analysis and diagnosis dataset^58^. There are 1272 dental radiographs in this dataset, which are divided into four labels: implants, cavities, fillings, and impacted teeth. Interestingly, specific images might display more than one classification simultaneously. The dataset has been preprocessed to simplify training a classifier for each class. The original images were cropped to produce distinct images for each distinct class. As a result, 4023 images were used for training, 402 for validation, and 392 for testing. Figure 6 indicated some images from the dataset with four labels: filling, implanted, impacted, and cavity.

The dental radiography dataset used in this study is a comprehensive collection of X-ray images specifically curated to aid in diagnosing various dental conditions such as fillings, implants, impacted teeth, and cavities. The dataset consists of 4023 images, classified into four distinct classes based on the aforementioned conditions. Each class has a representative number of images to ensure a balanced dataset, with 2609 images for fillings, 910 for implants, 301 for impacted teeth, and 203 for cavities.

The dataset was divided into training and testing sets using a cross-validation strategy to ensure a robust model evaluation. We used a 5-fold cross-validation approach, dividing the dataset into five subsets. Four subsets were used for training in each iteration, with the remaining subset set aside for testing. This process was repeated five times to ensure that each subset was tested only once. This strategy provided a reliable estimate of the model’s performance and reduced the risk of overfitting by exposing the model to a variety of data subsets during training.

### Evaluation metrics

Many evaluation metrics are used to analyze the performance of the dental diagnosis method. This section introduces the mathematical formulations which are used to compute these metrics. The initial measures are used for metrics calculation as true positive (TP) occurs when the classifier predicts an image with a dental diseases label has a disease, false positive (FP) occurs when the classifier predicts an image with no dental diseases label has a disease, true negative (TN) occurs when the classifier predicts an image with no dental diseases label has no disease. Besides, false negative (FN) occurs when the classifier predicts an image with a dental disease label has no disease. Some metrics are utilized for this evaluation, such as accuracy (ACC), precision (PRE), sensitivity (SEN), specificity (SPE), Dice similarity coefficient (DSC), and Matthews correlation coefficient (MCC) that can be computed using Eqs. (15)–(20), respectively.

15 $$\begin{aligned} ACC= & \frac{TP+TN}{TP+FP+TN+FN} \end{aligned}$$

16 $$\begin{aligned} PRE= & \frac{TP}{(TP+FP)} \end{aligned}$$

17 $$\begin{aligned} SEN= & \frac{TP}{(TP+FN)} \end{aligned}$$

18 $$\begin{aligned} SPE= & \frac{TN }{(TN + FP)} \end{aligned}$$

19 $$\begin{aligned} DSC= & 2\times \frac{PRE\times SEN}{PRE + SEN} \end{aligned}$$

20 $$\begin{aligned} MCC= & \frac{(TP * TN - FP * FN)}{\sqrt{((TP + FP) * (TP + FN) * (TN + FP) * (TN + FN))}} \end{aligned}$$

### Results

We performed an extensive evaluation process to determine the most advanced DL technique for diagnosing dental caries. Several pre-trained models, classifiers, and transformer architectures were systematically compared in experiments. We set out to surpass current approaches and determine the optimal strategy for precise caries detection by carefully evaluating every configuration.

To address the overfitting issue, we used several regularization techniques, such as dropout and early stopping, during model training. Dropout was used in various layers of the network to keep the model from relying too heavily on any one set of features, and early stopping was used to halt training once the model’s performance on the validation set plateaued, preventing overfitting to the training data.

Pre-trained models such as MobileNet^59^, Inception^60^, ResNet variants^61^, VGG architectures^62^, InceptionResNetV2^63^, DenseNet^64^, and EfficientNet^65^ were compared side by side in the first investigation. Using our dental caries dataset, each model was refined and evaluated. This preliminary investigation aimed to find the pre-trained model that best captured the characteristics that allow one to distinguish between teeth with caries and those with healthy ones. Table 2 provides a thorough analysis of the pre-trained model’s performance.

**Table 2:** Experimental results for various pre-trained models.

Model	PRE (%)	SEN (%)	SPE (%)	DSC (%)	ACC (%)	MCC (%)
EfficientNetB0	80.0	82.5	82.4	80.0	90.78	91.3
VGG16	95.0	78.5	79.8	84.8	92.5	91.2
VGG19	82.5	81.0	81.3	79.8	91.0	89.7
ResNet50	93.0	79.0	80.1	85.4	91.6	90.5
InceptionV3	80.5	83.5	83.0	81.5	91.3	91.2
DenseNet121	91.2	81.5	82.2	85.3	92.8	91.1
MobileNetv1	86.3	90.3	91.4	88.0	93.7	91.5
InceptionResNetV2	77.5	57.3	58.1	56.5	72.6	70.4

The evaluation of the pre-trained model found significant performance variations. Specific models, such as MobileNetv1 and InceptionResNetV2, performed better than others. This preliminary investigation emphasizes how crucial it is to choose the suitable pre-trained model for the best caries detection. After finding some attractive pre-trained models, we investigated further to find out how various classifiers affected the models’ overall performance. We used various classifiers, such as DTs, random forests, KNN, multi-layer perceptrons (MLP), and SVM with different kernels (linear, radial Basis function (RBF), and poly). These classifiers were paired with each pre-trained model and assessed the two’s performance. This experiment aimed to determine if any classifiers could significantly improve performance by complementing each pre-trained model’s ability to extract features. The ACC attained by several pre-trained models combined with distinct classifiers is shown in Table 3.

**Table 3:** Experimental results of various pre-trained DL models with different classifiers.

Model	Classifier name	PRE (%)	SEN (%)	SPE (%)	DSC (%)	MCC (%)	ACC (%)
EfficientNetB0	SVM(Linear)	90.5	82.5	81.8	86.0	89.1	92.8
	SVM (Poly)	85.0	73.0	75.4	75.0	74.2	89.87
	SVM (RBF)	86.0	74.0	73.7	77.0	77.4	91.56
	Decision Tree	62.0	61.0	63.2	61.0	60.5	78.5
	Random Forest	68.0	60.0	60.2	63.0	62.4	86.5
	MLP	85.5	81.5	83.0	83.5	83.2	91.5
	KNN	74.5	69.5	70.8	71.5	70.6	86.9
VGG16	SVM(Linear)	69.5	75.5	76.0	71.5	72.3	82.3
	SVM (Poly)	67.5	54.5	54.4	58.5	58.8	85.2
	SVM (RBF)	88.5	70.5	71.6	76.5	78.0	89.03
	Decision Tree	54.5	55.5	55.7	54.5	53.2	75.1
	Random Forest	93.5	61.5	60.9	67.5	65.8	86.9
	MLP	69.5	67.5	67.6	68.5	69.0	85.23
	KNN	75.5	64.5	66.0	68.5	67.4	83.1
VGG19	SVM(Linear)	60.5	64.5	63.8	62.5	61.3	78.1
	SVM (Poly)	78.5	62.5	62.3	67.5	65.8	88.6
	SVM (RBF)	88.0	73.0	73.5	78.0	78.6	90.7
	Decision Tree	65.5	56.5	57.1	59.5	58.6	77.6
	Random Forest	95.0	59.0	61.2	66.0	66.1	86.9
	MLP	79.5	73.5	74.8	75.5	73.6	88.2
	KNN	70.5	59.5	60.0	63.5	62.0	83.1
ResNet50	SVM(Linear)	85.5	85.5	85.5	85.5	86.1	90.3
	SVM (Poly)	88.5	74.5	75.0	79.5	77.8	90.7
	SVM (RBF)	92.5	75.5	76.3	80.5	78.6	90.7
	Decision Tree	56.0	60.0	62.1	58.0	59.2	70.9
	Random Forest	82.5	62.5	62.5	68.5	68.2	86.1
	MLP	83.5	82.5	83.3	82.5	80.5	90.7
	KNN	77.5	72.5	72.7	73.5	72.0	86.9
InceptionV3	SVM(Linear)	66.5	65.5	66.0	65.5	65.1	81.0
	SVM (Poly)	83.5	60.5	60.3	64.5	64.1	83.5
	SVM (RBF)	71.5	60.5	61.5	64.5	65.2	83.1
	Decision Tree	56.5	57.5	57.6	56.5	56.0	72.6
	Random Forest	80.5	59.5	61.1	64.5	63.7	83.5
	MLP	70.5	61.5	61.5	64.5	62.9	84.4
	KNN	71.5	62.5	63.8	65.5	66.1	82.7
DenseNet121	SVM(Linear)	74.5	75.5	76.4	74.5	74.0	84.4
	SVM (Poly)	59.5	53.5	53.5	55.5	55.2	80.2
	SVM (RBF)	91.5	72.5	73.3	79.5	78.1	87.34
	Decision Tree	53.5	53.5	53.6	52.5	52.0	71.3
	Random Forest	92.5	68.5	68.8	75.5	73.4	86.1
	MLP	83.5	81.5	82.4	82.5	82.4	89.1
	KNN	79.5	71.5	71.6	75.5	76.1	84.4
MobileNetV1	SVM(Linear)	60.0	62.0	62.3	60.9	61.1	64.9
	SVM (Poly)	61.0	61.5	61.5	61.7	60.8	65.0
	SVM (RBF)	60.0	62.0	61.8	61.8	60.0	64.9
	Decision Tree	62	60	61.3	61.8	61.5	64.9
	Random Forest	63.0	59.0	59.7	61.5	61.6	64.5
	MLP	63.0	58.5	59.3	61.7	61.5	64.5
	KNN	60.0	62.0	62.3	61.8	61.6	64.8
InceptionResNetV2	SVM(Linear)	57.0	45.0	46.5	46.0	45.8	75.9
	SVM (Poly)	39.0	36.0	36.1	36.0	35.5	73.8
	SVM (RBF)	36.0	34.0	34.3	33.0	33.0	71.3
	Decision Tree	37.5	38.5	38.7	37.0	36.9	61.6
	Random Forest	45.0	41.0	41.5	42.0	42.0	70.5
	MLP	36.0	33.0	33.2	34.4	33.6	60.4
	KNN	49.0	39.0	39.3	41.0	40.5	69.2

The evaluation of pre-trained models using different classifiers (Table 3) showed that the choice of classifier significantly affected overall ACC. Specific models, like EfficientNetB0, worked well with classifiers like SVM. This emphasizes the importance of investigating how classifiers and pre-trained models interact for the best caries detection.

The possibility of combining vision transformers with the pre-trained models that were the most successful in the earlier phases was investigated in the following phase. We thought the ACC of caries detection could be further improved by the vision transformer’s capacity to recognize long-range dependencies within dental images. The ensemble models that resulted from combining each pre-trained model with a vision transformer were assessed using the same set of classifiers that were used previously. This experiment revealed which classifier best utilized the combined power of both architectures and whether vision transformers provided an additional performance gain over using pre-trained models alone.

Interesting outcomes were obtained when different pre-trained deep learning models were integrated with vision transformers. We aimed to potentially improve caries detection ACC by utilizing the vision transformer’s capacity to learn long-range dependencies within dental images. The performance of these combined models when combined with various classifiers is displayed in Table 4.

**Table 4:** Experimental results of various pre-trained DL models combined with vision transformer based on different classifiers.

Model	Classifier name	PRE (%)	SEN (%)	SPE (%)	DSC (%)	MCC (%)	ACC (%)
EfficientNetB0+Vit	SVM (Linear)	88.5	81.5	81.7	83.5	83.1	92.8
	SVM (Poly)	92.0	69.0	70.3	71.0	72.2	89.8
	SVM (RBF)	93.5	76.5	76.7	79.5	80.0	92.4
	Decision Tree	62.0	63.0	63.3	62.0	61.5	74.3
	Random Forest	95.0	69.0	69.1	76.0	76.7	88.6
	MLP	91.0	79.0	80.0	81.0	81.4	92.4
	KNN	70.5	64.5	64.0	67.5	67.0	84.8
VGG16+Vit	SVM (Linear)	69.5	75.5	75.6	72.5	73.0	82.3
	SVM (Poly)	68.8	55.5	56.2	59.0	58.3	86.0
	SVM (RBF)	89.5	71.0	71.6	77.0	78.1	89.03
	Decision Tree	53.0	54.0	54.2	53.3	53.0	71.7
	Random Forest	84.0	65.0	65.3	71.0	71.4	82.7
	MLP	77.5	70.7	70.5	73.0	72.7	86.9
	KNN	75.8	65.0	64.8	69.0	68.5	83.1
VGG19+Vit	SVM (Linear)	61	64.3	64.2	62.5	62.5	78
	SVM (Poly)	79.3	60.5	60.5	67.5	67.2	88.6
	SVM (RBF)	87.8	72.8	72.7	77.8	78.0	90.7
	Decision Tree	57.3	56.5	56.6	56.3	56.0	72.2
	Random Forest	82	67	67.2	72	72.1	84.4
	MLP	73.5	68	67.8	70	70.3	85.7
	KNN	71.2	59.8	60	63.8	63.5	83.1
Resnet50+Vit	SVM (Linear)	85.5	85.5	85.5	85.5	86	90.3
	SVM (Poly)	89	72.7	72.5	78	78.1	89.9
	SVM (RBF)	92.5	75.7	75.4	80.6	80.5	90.7
	Decision Tree	62.5	65	65.3	64	63.8	77.2
	Random Forest	93	70	70.2	76	75.6	88.2
	MLP	86	84	84.1	85	85	91.6
	KNN	77.5	72.5	72.5	73.5	73.2	86.9
InceptionV3+Vit	SVM (Linear)	66.5	65.5	65.5	66	66.2	81.1
	SVM (Poly)	83.5	58.7	80	64	63.8	83.5
	SVM (RBF)	72	61	61.2	65	65	83.1
	Decision Tree	61	59	59.3	60	60.2	75.1
	Random Forest	85.5	62.5	62.5	63	63.2	84.8
	MLP	63.5	61.5	61.3	62.7	62	82.3
	KNN	71.7	63	63.1	65.5	65.1	82.7
DenseNet121+Vit	SVM (Linear)	73.5	74.5	74.6	73.5	73.2	84.4
	SVM (Poly)	62.5	53	53.3	56	56	80.6
	SVM (RBF)	90.5	71.5	71.5	78.5	78	87.3
	Decision Tree	67.5	61.5	61.7	63	63.4	72.6
	Random Forest	86.5	68	67.8	73.5	73.5	83.5
	MLP	85.5	77.5	77.4	81	81.1	88.6
	KNN	79	71	71.3	75	75	84.4
MobileNetV1+Vit	SVM (Linear)	63	62.5	63	62.7	62.5	64.9
	SVM (Poly)	62.7	62	62.3	62.5	62.2	64.9
	SVM (RBF)	62.7	63	63	62.5	62.5	64.9
	Decision Tree	59	56	56.2	56	55.8	68.8
	Random Forest	80.5	62.5	62.4	67.5	67.3	78.5
	MLP	70	61	61.4	65.2	65	73.4
	KNN	59	56	56	56	55.6	68.7
InceptionResNetV2+Vit	SVM (Linear)	59	56	56.1	56	56	75.1
	SVM (Poly)	59.5	63.8	63.7	62.7	62.5	70.5
	SVM (RBF)	59	56	56.2	56	55.6	64.9
	Decision Tree	57.5	59.5	59.6	57.5	57.4	73.4
	Random Forest	78.5	58.5	58.5	61.5	61.2	87.9
	MLP	59	56	56	56	55.5	64.9
	KNN	57.5	58.5	58.7	58	58	69.2

We looked into Swin Transformers, which is well-known for its effectiveness and reliable feature learning. Each pre-trained model was integrated with a Swin Transformer and assessed using a variety of classifiers, much like in the vision transformer experiment. This last phase aimed to determine whether Swin Transformers could outperform both vision transformer combinations and standalone pre-trained models. The results showed that the best ACC in caries classification was obtained when the Swin Transformer was combined with MobileNet and bagging classifier. By utilizing each component’s strengths to its fullest, this configuration pushed the limits of dental caries diagnosis accuracy.

As shown in Table 5, highly encouraging results were obtained. The remarkable ACC of 95.6% was attained by combining the Swin Transformer with the MobileNet pre-trained model and an SVM classifier with a linear kernel. This configuration outperformed all other combinations investigated in this study. A better method for classifying dental caries was produced by combining the strength of the SVM’s decision-making abilities with the long-range dependency learning of Swin Transformer and the efficiency of MobileNet. This result emphasizes how Swin Transformers can be used to push the limits of accuracy in tasks involving medical image analysis.

**Table 5:** Experimental results of various pre-trained DL models combined with Swin Transformer based on different classifiers.

Model	Classifier name	PRE (%)	SEN (%)	SPE (%)	DSC (%)	MCC (%)	ACC (%)
EfficientNetB0 + Swin	SVM (Linear)	94.0	94.0	94.2	93.0	92.8	92.4
	SVM (Poly)	91.6	91.4	91.5	90.2	90.4	91.1
	SVM (RBF)	90.6	90.6	91.0	89.6	89.8	90.5
	Decision Tree	74.6	75.0	75.2	74.8	74.5	75.1
	Random Forest	85.4	86.8	86.7	86.1	86.3	86.6
	MLP	94.2	94.2	94.2	93.2	93.0	93.2
	XGBoost	90.6	90.8	91.0	90.7	90.5	90.8
	KNN	85.6	85.2	85.5	85.3	86.0	85.6
	Bagging Classifier	92.1	92.5	92.7	92.4	92.8	93.2
VGG16 + Swin	SVM (Linear)	88.0	88.9	88.8	88.3	89.1	89.7
	SVM (Poly)	82.1	84.3	84.2	83.2	83.0	86.7
	SVM (RBF)	82.9	85.3	85.7	83.6	83.1	85.3
	Decision Tree	69.7	67.2	67.2	68.4	68.0	70.9
	Random Forest	77.0	78.3	79.0	77.7	77.5	80.9
	XGBoost	87.2	86.5	86.3	86.4	85.7	85.7
	MLP	89.3	89.4	89.2	89.3	89.5	86.8
	KNN	80.0	79.1	79.5	79.5	79.1	79.3
	Bagging Classifier	89.3	88.9	89.1	88.1	87.3	90.9
VGG19 + Swin	SVM (Linear)	91.4	90.8	91.0	90.6	90.5	90.6
	SVM (Poly)	86.0	87.8	87.8	85.4	86.2	87.4
	SVM (RBF)	85.8	88.2	88.0	87.1	86.5	87.8
	Decision Tree	73.8	73.8	73.6	73.4	73.1	73.53
	Random Forest	84.2	84.0	84.2	84.1	84.0	84.0
	MLP	92.4	92.2	92.2	91.6	91.3	90.9
	XGBoost	89.2	89.8	89.5	88.6	89.1	89.5
	KNN	80.2	80.2	80.3	79.2	80.0	80.3
	Bagging Classifier	91.2	91.0	91.1	91.1	91.0	91.5
Resnet50 + Swin	SVM (Linear)	92.4	93	93.2	92.8	92.5	92.9
	SVM (Poly)	93.2	93.2	93.2	92.2	92.5	92.9
	SVM (RBF)	95.0	95.0	95.2	94.0	92.5	91.9
	Decision Tree	76.4	76.4	76.7	75.4	75.8	76.18
	Random Forest	79.6	86.4	86.5	84.4	85.0	86.43
	MLP	94.8	94.6	95.1	94.4	93.8	93.73
	XGBoost	91.8	90.6	90.5	91.4	91.3	91.9
	KNN	87.8	87.0	87.2	86.0	86.4	87.1
	Bagging classifier	91.7	92.3	92.5	92.0	92.3	93.7
InceptionV3 + Swin	SVM (Linear)	91.0	90.8	91.0	90.6	90.8	90.7
	SVM (Poly)	83.2	87.6	86.8	84.4	85.0	87.46
	SVM (RBF)	82.8	86.8	86.8	83.4	84.2	86.6
	Decision Tree	71.2	71.0	71.5	70.8	70.5	71.1
	Random Forest	80.2	82.0	82.4	80.0	80.3	82.1
	MLP	91.0	90.6	91.5	90.7	90.5	90.6
	XGBoost	87.8	87.4	87.5	86.2	86.5	87.3
	KNN	83.8	82.2	82.2	81.6	81.5	82.3
	Bagging classifier	91.0	90.7	90.8	90.9	91.0	91.6
DenseNet121 + Swin	SVM (Linear)	93.2	93.2	93.5	93.2	93.2	92.3
	SVM (Poly)	89.8	91.0	91.7	89.2	88.5	90.9
	SVM (RBF)	88.8	90.6	90.7	88.4	89.6	90.6
	Decision Tree	78.0	77.0	77.7	77.6	77.5	77.3
	Random Forest	86.2	86.0	86.3	84.4	84.7	86.1
	MLP	94.2	94.2	95.3	95.2	95.8	93.2
	XGBoost	91.6	91.8	92.3	91.0	91.5	91.7
	KNN	84.2	83.8	83.6	82.0	82.8	83.7
	Bagging classifier	94.0	93.6	93.5	93.8	93.7	93.4
**MobileNetV2 + Swin**	SVM (Linear)	94.2	94.2	94.2	94.2	94.3	94.3
	SVM (Poly)	93.9	93.2	93.1	92.7	93.0	92.9
	SVM (RBF)	92.2	91.9	92.3	90.8	91.1	91.4
	Decision Tree	73.8	73.5	73.8	73.6	73.5	73.7
	Random Forest	87.2	86.2	86.5	84.8	85.2	85.1
	MLP	92.0	91.9	92.2	91.9	92.0	93.8
	XGboost	91.7	91.5	91.8	90.8	90.6	91.8
	KNN	86.1	85.3	85.5	84.0	84.7	85.1
	Bagging classifier	**95.7**	**95.4**	**95.7**	**95.5**	**95.3**	**95.6**
InceptionResNetV2+Swin	SVM (Linear)	88.0	88.0	88.3	88.0	87.5	87.7
	SVM(Poly)	85.0	87.0	87.2	85.9	86.1	86.8
	SVM(RBF)	83.0	86.0	86.3	84.4	85.2	85.9
	Decision Tree	69.8	69.0	69.6	69.4	69.1	69.2
	Random Forest	79.6	81.2	81.1	80.4	81.3	80.8
	MLP	90.0	89.0	89.1	89.4	88.9	89.37
	XGBoost	85.4	85.0	85.1	85.2	85.1	85.0
	KNN	78.0	78.6	78.7	75.8	76.3	78.6
	Bagging classifier	88.2	88.7	89.1	88.4	89.0	88.9

Significant values are given in bold.

Our model diagnoses various dental conditions, including cavities, fillings, implants, and impacted teeth. Each condition requires the model to focus on different aspects of the X-ray images-such as fine detail recognition for cavities and broader context understanding for implant placement. By averaging the outputs of multiple base classifiers, bagging enhances the model’s ability to generalize across these different tasks. This is particularly beneficial when dealing with imbalanced data, where some conditions may be underrepresented in the training set. The bagging ensemble mitigates the risk of the model being biased toward more frequent conditions, ensuring a more balanced performance across all classes.

We aimed to determine our dataset’s most accurate and dependable method for dental caries detection by thoroughly assessing different pre-trained models, classifiers, and transformer combinations. Analyzing these experimental results identified a clear front-runner for clinical applications, especially the Swin Transformer’s remarkable performance with MobileNet and the bagging classifier. This configuration outperformed all other combinations tested, achieving a remarkable ACC of 95.6% (see Table 5 for specific results). The Hyperparameters for Swin Transformer and MobileNetV2 Models are shown in Table 4.

**Table 6:** The used hyperparameters for Swin Transformer and MobileNetV2 models.

Hyperparameter	Swin Transformer	MobileNetV2
Learning rate	0.00001	0.0001
Optimizer	AdamW	Adam
Weight decay	0.01	0.0001
Batch size	32	32
Patch size	$4\times 4$	N/A
Window size	$7\times 7$	N/A

**Figure 7:**
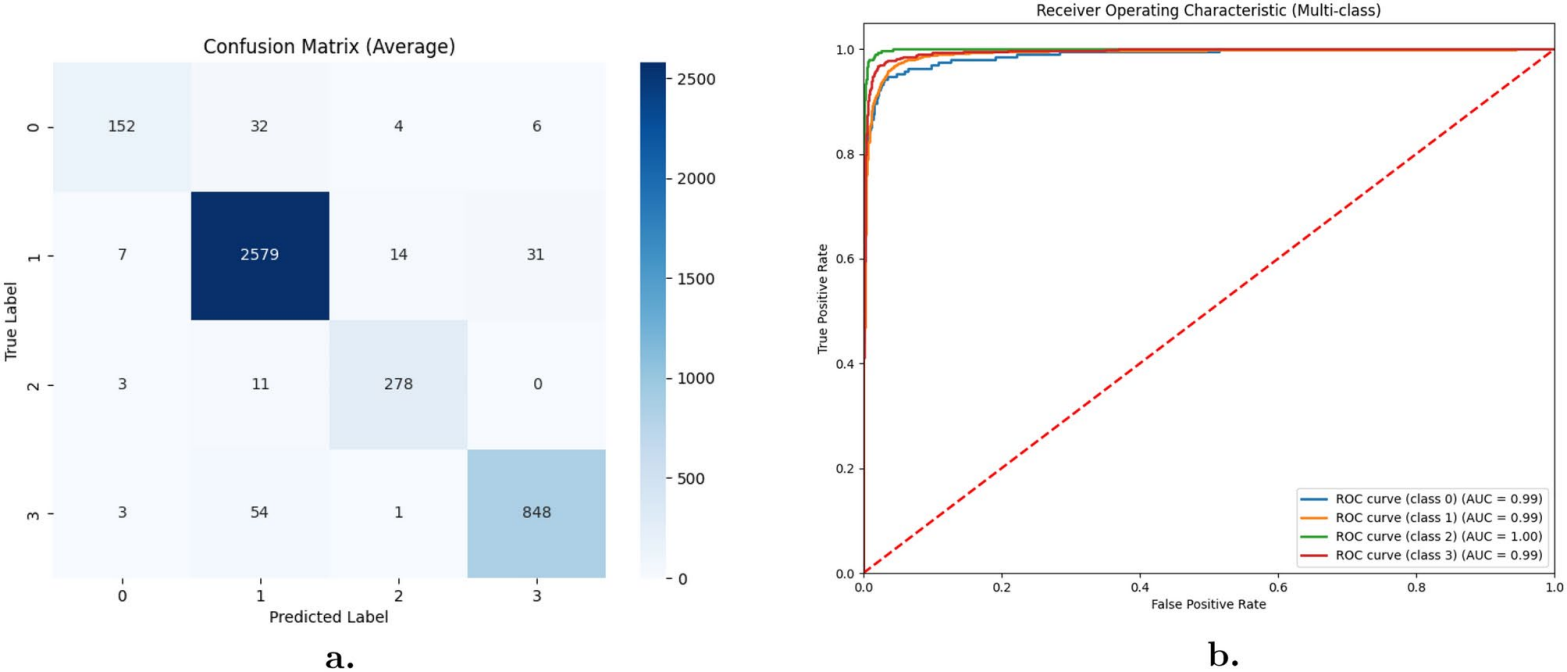
(**a**) Confusion matrix for dental radiography analysis and diagnosis dataset. (**b**) ROC curve for dental radiography analysis and diagnosis dataset.

A 5-fold cross-validation strategy was used to comprehensively evaluate our model’s performance on the dental radiography analysis and diagnosis dataset. The confusion matrix is produced using this method, which graphically represents the model’s radiograph classification (Fig. 7). The model’s performance in identifying each distinct dental abnormality in the dataset is shown in Table 5, which provides a class-specific breakdown. This model makes possible enhanced evaluation of the model’s generalizability and efficacy in dental radiograph analysis in the real world.

**Table 7:** The experimental results for each class.

Class name	ACC	PRE	SEN	SPE	DSC	MCC
Implant	93.7	91.3	91.4	91.2	90.8	90.6
Fillings	96.8	96.3	96.4	97.2	96.8	96.5
Impacted tooth	96.7	95.6	95.1	95.3	95.4	95.5
Cavity	96.1	94.6	93.7	94.1	94.5	94.3

We have thoroughly compared our suggested approach and several cutting-edge (SOTA) methods for diagnosing dental diseases. A thorough comparison is given in Table 6, which also highlights the methodologies and performance metrics used in the various studies. Our approach performs competitively in the diagnosis of dental diseases. It combines a hybrid MobileNetV2 and Swin Transformer for feature extraction with a bagging classifier for classification. More specifically, we outperformed multiple SOTA methods with an average accuracy of 96.5%.

**Table 8:** The comparison of proposed hybrid model with the state-of-the-art methods.

Study	Method	Performance
Deng et al.^66^	Customized CNN architecture	ACC: 93.04%
Abdalla-Aslan et al.^67^	segmented using adaptive threshold, Gray level values and shape features, Cubic SVM with Error-Correcting Output Codes for classificatiob	ACC: 93.6%
Ghaznavi et al.^68^	CNN comparison with AlexNet and VGGNet16	Average PRE: 92%
Jaiswal et al.^69^	Transfer learning (ResNet, MobileNet) + XGBoost	average ACC: 93%
Rajee and Mythili^70^	Inception ResNetV2	ACC: 94.51%
**Proposed hybrid model**	**Hybrid approach (MobileNetV2 + Swin Transformer) with bagging ensemble**	ACC: 96.5

### Discussion

The performance of several pre-trained deep learning models, classifiers, and their combinations for dental radiograph analysis using our suggested framework is examined in this section, which also explores our experimental results. We compared and contrasted Inception, MobileNet, ResNet variations, VGG architectures, InceptionResNetV2, DenseNet, and EfficientNet, as well as vision and Swin Transformers, which are used for feature extraction and paired with various classifiers. We examined how well different pre-trained models performed in classifying dental caries, as shown in Table 2. Important information about how well each model extracts discriminative features from dental images was obtained from this analysis. The most reliable method for diagnosing caries will be determined by analyzing the data in more detail and combining it with results from later tests utilizing transformer architectures and classifiers.

For pre-trained models in dental radiograph classification, our experiments showed a trade-off between computational efficiency and accuracy. Though their ability to capture local details effectively allowed models like ResNet variants and VGG architectures to achieve strong performance, they can be computationally expensive. The lightweight model MobileNet, on the other hand, showed a good balance between efficiency and accuracy. We used MobileNet as the pre-trained model for feature extraction in our framework. This decision was made to maintain computational efficiency and achieve good classification accuracy-a critical goal for real-world deployment scenarios where processing time may be limited-with that goal in mind. Notably, although MobileNet may not fully capture all the fine details compared to more complex models, its computational efficiency was a significant advantage, and its performance was adequate for our task.

To achieve optimal caries detection performance, the interplay between classifiers and pre-trained models emerged as a crucial factor. The results underscore the significance of closely assessing these interactions, even though specific models, such as EfficientNetB0, demonstrated robust synergy with classifiers like SVMs, as shown in Table 3. Our ability to accurately diagnose caries and analyze dental radiographs can be fully realized by exploring this relationship in greater detail.

In classifying dental diseases, a hybrid approach has definite advantages, as our investigation of various architectures has shown. In terms of capturing local details indicative of dental abnormalities, evaluating pre-trained CNN models alone produced promising results (Table 2). But by providing useful decision-making boundaries inside the high-dimensional feature space produced by the CNN, adding a machine learning classifier, such as an SVM, enhanced performance even more (Table 3). The classifier selection had a significant influence on the overall performance of our proposed method. Our research shows that SVMs performed comparably to other classifiers when implemented in our framework. This makes sense because SVMs are good at handling high-dimensional feature spaces, and fused representations produced by combining transformers with pre-trained models are also fused representations.

The use of MobileNetV2 and Swin Transformer in our dual-stream approach has resulted in significant improvements in feature extraction and classification accuracy for dental disease diagnosis, particularly for fillings, implants, impacted teeth, and cavities. The hybrid model effectively combines the strengths of both architectures, yielding a more comprehensive feature representation that significantly improves diagnostic performance.

When vision transformers and Swin transformers were combined with pre-trained models, the classification accuracy was significantly higher than when pre-trained models were used exclusively (Tables 4, 5). This demonstrates how the transformers can extract contextual information and long-range dependencies from dental radiographs, enhancing the local detail extraction capabilities of pre-trained models. We can also determine which classifier makes the most use of the advantages of both architectures, which could result in a notable improvement in the accuracy of caries classification. This investigation opens the door to more research on the best arrangement for combining vision transformers with trained models for more accurate dental caries diagnosis.

The MobileNetV2 component of our model excelled at capturing intricate local details in dental X-ray images, such as precise cavity boundaries and detailed structural integration of dental implants. These local features are critical for accurately identifying small-scale abnormalities, which may be overlooked by models that only consider larger image contexts. On the other hand, the Swin Transformer successfully captured global contextual information, such as the spatial relationships between impacted teeth and neighboring structures and the overall placement of implants within the dental arch. This dual-stream extraction ensures that both micro and macro-level features are accurately represented, resulting in a more robust and reliable diagnosis.

Interestingly, when compared to vision transformers, Swin Transformers continuously produced better performance. This might be explained by the hierarchical architecture of Swin Transformers, which enables them to learn features at various scales and produce a richer representation for the classification of diseases. This research demonstrates the potential of Swin Transformers for superior feature learning in conjunction with the effectiveness of MobileNet and the solid decision-making of SVM, opening the door for a significant improvement in dental care through more accurate caries diagnosis.

In terms of classification accuracy, the hybrid model consistently outperformed single-stream approaches based solely on MobileNetV2 or Swin Transformer. Specifically, the hybrid model outperformed other models in detecting cavities and distinguishing between implants and impacted teeth. This improvement is due to the complementary nature of the features extracted by both streams, which improves the model’s ability to distinguish between similar conditions and lowers misclassification rates.

When compared to a MobileNetV2-only model, the hybrid approach performed better in situations where global context was important, such as identifying impacted teeth and assessing implant placement. The Swin Transformer’s global context understanding helped to reduce errors caused by a lack of broader image context in the MobileNetV2-only model, as illustrated in Table 7.

**Table 9:** The performance comparison of the MobileNetV2-Only model and the hybrid model (MobileNetV2 + Swin Transformer).

Task	Metric	MobileNetV2-only model	Hybrid model (MobileNetV2 + Swin Transformer)	Improvement
Identifying impacted teeth	ACC	89.4%	96.7%	+7.2%
	PRE	88.3%	95.6%	+7.3%
	SEN	87.6%	95.1%	+7.5%
	DSC	88.0%	95.4%	+6.4%
Assessing implant placement	ACC	91.0%	96.8%	+5.8%
	PRE	90.2%	96.3%	+6.1%
	SEN	87.1%	93.7%	+6.6%
	DSC	90.4%	96.4%	+6.0%

Similarly, when compared to a Swin Transformer-only model, the hybrid approach performed significantly better in diagnosing conditions requiring fine detail recognition, such as identifying small cavities or accurately delineating fillings. The MobileNetV2 component provided a level of precision that the Swin Transformer, with its broader focus, could not match, as illustrated in Table 8.

**Table 10:** The comparison of the performance metrics for the hybrid model and the Swin Transformer-only model.

Task	Metric	Swin Transformer-only model	Hybrid model (MobileNetV2 + swin transformer)	Improvement
Identifying small cavities	ACC	89.6%	96.1%	+6.5%
	PRE	88.6%	94.6%	+6.0%
	SEN	87.1%	93.7%	+6.6%
	DSC	88.1%	94.5%	+6.4%
Delineating fillings	ACC	91.0%	96.8%	+5.8%
	PRE	90.2%	96.3%	+6.1%
	SEN	87.1%	93.7%	+6.6%
	DSC	90.4%	96.4%	+6.0%

Using the bagging classifier significantly improves the robustness of our hybrid model. Combining predictions from different classifiers reduces the possibility of overfitting to a specific subset of the data. This is especially important in our study, where various dental diseases, such as cavities, fillings, implants, and impacted teeth, necessitate a model that can generalize well across multiple cases. The bagging ensemble improves accuracy and reliability by leveraging the diversity of its base classifiers to handle a wide range of dental conditions.

When working with complex datasets like dental radiographs, where overfitting can be an issue because of the minute differences in features like fillings and cavities, bagging’s ability to reduce overfitting is especially helpful. Bagging effectively reduces the risk of overfitting the training data by training multiple base classifiers on different bootstrap samples and aggregating their predictions.

On the other hand, techniques such as XGBoost, which concentrate on successively boosting weak classifiers, may be more vulnerable to overfitting, particularly in cases where the base models lack proper regularization. XGBoost and other boosting algorithms try to fix mistakes made by earlier models in the sequence. This can occasionally result in extremely complex models that fit the training data too closely.

While the bagging ensemble method offers several advantages, it is important to acknowledge its limitations in this context. One potential drawback is the computational overhead of training multiple base classifiers, which can be resource-intensive, particularly when working with large datasets or complex models. Additionally, while bagging effectively reduces variance, it may not always provide the same level of performance gains as boosting methods, which are designed to reduce bias and variance by focusing on difficult-to-classify instances.

We reduced the computational load by utilizing parallel processing and effective libraries. For example, we accelerated the training process by utilizing hardware acceleration and multi-threading. We used cross-validation and performance metrics to continuously monitor and validate our ensemble model’s performance. By using this procedure, we detected any possible problems early on and modified the ensemble configuration to meet the required performance standards.

The proposed model’s integration into clinical workflows represents a promising advance in dental disease diagnosis. By combining the strengths of MobileNetV2 and Swin Transformer, the model can help dental professionals diagnose conditions such as fillings, implants, impacted teeth, and cavities more accurately and efficiently. This dual-stream approach enables the model to process fine details and larger image contexts, making it ideal for clinical settings requiring quick and accurate decision-making. Integrating this model into existing imaging systems could improve diagnostic accuracy while reducing analysis time, ultimately improving patient outcomes.

Deploying this AI-driven model in real-world clinical settings presents some challenges that must be addressed to ensure its effectiveness and dependability. Data privacy is a significant concern, especially when handling sensitive patient information. Compliance with healthcare regulations, such as HIPAA, is critical to the widespread adoption of this technology. Furthermore, the model must be adaptable to the various imaging equipment and techniques used in different dental practices. This variability can impact the model’s performance, necessitating regular updates and retraining on various datasets to ensure accuracy. Gaining acceptance from dental professionals will also be critical, which may necessitate rigorous validation of the model’s effectiveness in various clinical settings.

Integrating AI in healthcare, particularly diagnostic tools like the proposed model raises important ethical considerations. Ensuring transparency in AI decision-making is essential to build trust among clinicians and patients. The model should provide interpretable results, allowing healthcare providers to understand and explain the AI’s diagnoses to patients. Additionally, there is a need to safeguard the patient-clinician relationship, ensuring that the introduction of AI does not undermine the trust and communication critical to adequate healthcare.

The current model demonstrates significant potential for enhancing dental disease diagnosis, but several avenues for future research could further improve its applicability and effectiveness. One key area is improving the model’s generalization to different patient populations and imaging modalities, such as 3D dental scans or intraoral images. Additionally, future work could focus on integrating this model with other AI-driven diagnostic tools to create a more comprehensive system capable of addressing a wider range of dental and medical conditions. Addressing potential biases in the model’s predictions, particularly those related to demographic factors, will also be critical to ensuring that the benefits of AI-driven diagnostics are equitably distributed.

## Conclusion

In this study, we presented a new DL model that uses dental X-ray imaging to diagnose dental diseases. Our framework uses a hybrid approach to overcome the shortcomings of individual models. This creates a more reliable method for classifying dental diseases from radiographs by combining the power of CNNs in local detail extraction with the global context-capturing capabilities of transformers. While the Swin Transformer gathers the long-range dependencies necessary for precise disease classification, the MobileNetV2 backbone effectively extracts spatial features from the X-ray images. Finally, a bagging classifier offers strong decision boundaries for the identification of diseases. Evaluation results showed encouraging disease classification performance, demonstrating the complementary nature of transformer-based long-range dependency learning and MobileNet’s efficiency. In order to improve the model’s diagnostic abilities, future research will investigate adding other modalities, such as clinical data. Also, future work will include adding severity classification capabilities and broadening our model scope to accommodate a greater variety of dental abnormalities. Moreover, investigating attention-based feature fusion methods may help to improve feature combinations even more, resulting in feature representations that are even more precise and instructive.

## Data Availability

The datasets used during the current study available online at https://www.kaggle.com/datasets/imtkaggleteam/dental-radiography/data.
